# Risk factors for postoperative urinary retention in fragility hip fracture patients: a prospective study

**DOI:** 10.1186/s12877-024-05101-2

**Published:** 2024-06-03

**Authors:** Direk Tantigate, Nathanan Jansatjawan, Nath Adulkasem, Patkawat Ramart, Kongkhet Riansuwan

**Affiliations:** 1 Department of Orthopaedic Surgery, Faculty of Medicine Siriraj Hospital, Mahidol University, 2 Wanglang Road, Bangkoknoi, Bangkok, 10700 Thailand; 2 Division of Urology, Department of Surgery, Faculty of Medicine Siriraj Hospital, Mahidol University, Bangkok, Thailand

**Keywords:** Hip fracture, Urinary retention, POUR, Older, Screening tool

## Abstract

**Background:**

Postoperative urinary retention (POUR) among older patients with hip fractures is common and may result in delayed ambulation, prolonged hospital stays, and urinary tract infections. Although preoperative urinary catheter indwelling and early postoperative removal can prevent perioperative urinary retention, this condition may occur in some patients after catheter removal, which requires urinary catheter re-indwelling or intermittent catheterization. Therefore, this study aims to identify risk factors and develop a screening tool for postoperative urinary retention in patients who have undergone operative treatment for fragility hip fractures subsequent to urinary catheter removal.

**Methods:**

A prospective cohort study of 145 fragility hip fracture in older patients who were operatively treated between September 2020 and May 2022 was conducted. All patients were evaluated for urine retention after urinary catheter removal using a bladder scan. In addition, factors related to urinary retention were collected and utilized for screening tool development.

**Results:**

Of the included patients, 22 (15.2%) were diagnosed with POUR. A multivariable logistic regression model using a stepwise backward elimination algorithm identified the current use of drugs with anticholinergic effect (OR = 11.9, *p* = 0.012), international prostate symptom score (IPSS) ≥ 8 (OR = 9.3, *p* < 0.001), and inability to independently get out of bed within 24 h postoperatively (OR = 6.5, *p* = 0.051) as risk factors of POUR. The screening tool that has been developed revealed an excellent performance (AuROC = 0.85, 95%CI 0.75 to 0.91) with good calibration and minimal optimism.

**Conclusions:**

Current use of drugs with anticholinergic effects, IPSS ≥ 8, and inability to independently get out of bed within 24 h postoperatively are significant variables of POUR. For additional external validation, a proposed scoring system for POUR screening was developed.

**Trial registration:**

The study protocol was retrospectively registered in The Thai Clinical Trials Registry (TCTR20220502001: 2 May 2022).

## Background

Fragility hip fracture is one of the most common fractures in the older, with an incidence of 7 per 100,000 [1]. Operative treatment has been advocated in order to allow the patients to ambulate early and minimize potential complications from immobilization [2]. Although mobilization can be accomplished after the surgery, up to 50% of the surgical cases might encounter postoperative urinary retention (POUR), which eventually leads to urinary tract infection and prolonged hospitalization [3, 4]. Such problems can be prevented by perioperative urinary catheterization.

However, the appropriate time for catheter removal remains a challenging topic to be determined. Prolonged catheterization increases the risk of urinary tract infection [5]. On the other hand, too early removal might result in urinary retention. Several risk factors influencing POUR have been identified, including male gender, type of anesthesia, postoperative pain, medications, and comorbidities [6]. Nevertheless, the specific risk of POUR in fragility hip fracture patients has not been widely proposed.

Therefore, we aimed to identify the risk factors of POUR in geriatric hip fracture patients. In addition, we intended to develop a screening tool to identify the patients who may benefit from delayed urethral catheter removal in order to prevent urinary retention after catheter removal.

## Methods

### Study design and population

We conducted a prospective cohort study of fragility hip fracture patients who underwent operative treatment between September 2020 and May 2022. The study protocol complied with the Declaration of Helsinki and was approved by the institutional review board (131/2563). The study was retrospectively registered in The Thai Clinical Trials Registry (TCTR20220502001: 2 May 2022) and reported according to the STROBE guidelines. A total of 328 eligible patients were identified, with only one patient declining participation. All admitted patients consented to treatment, and the authors enrolled eligible patients post-admission. Fifty-four patients were excluded based on the following criteria: 32 required urine monitoring beyond 48 h postoperatively, 13 sustained high-energy trauma, multiple injuries or fractures, 7 had a history of pelvic radiation, and 1 had an indwelling Foley catheter before admission. Additionally, 131 patients were not enrolled during a period of time when a bladder scan was out of service.

### Treatment protocol and data collection

After informed consent to participate in this study was completed, the patient’s demographic information, including age, sex, body mass index (BMI), comorbidities, pre-injury ambulatory status, history of previous pelvic organ surgery, lower urinary tract symptoms (LUTS), and underlying urological conditions were recorded. LUTS was assessed by using the International Prostate Symptom Scores (IPSS), which can be applied for all genders [7, 8].

All patients were scheduled for operative treatment (fracture fixation and hip replacement) within 72 h after admission according to the hospital treatment guideline. Preoperative urethral catheterization was immediately indwelled and was removed at 48 h postoperatively to prevent perioperative urinary retention according to the hospital protocol. Perioperative data including anesthetic method, operative time, estimated intraoperative blood loss, opioid consumption, postoperative pain measured by the Visual Analog Scale (VAS), current use of drugs with anticholinergic effect, and postoperative ambulation were recorded.

### Postoperative urinary retention (POUR) assessment

Residual urine was measured after Foley catheter removal using an ultrasound bladder scanner (VitaScan PD®, Vitacon US LLC, Wayzata, MN, USA). We defined POUR as one of the following: (1) an inability to voluntarily void urine within 6 h, (2) a residual urine of equal or greater than 300 ml within 6 h, or (3) a residual urine of equal or greater than 300 ml two times within 24 h [9]. The urethral catheter was immediately re-indwelled in patients who were diagnosed with POUR.

### Statistical analysis

The mean, standard deviation (SD), and median with interquartile range (IQR) were used to demonstrate normal and non-normally distributed continuous data, respectively. Counts and percentages were used to present categorical data. Differences between patients who experienced POUR were tested using a t-test, Mann-Whitney U test, and Fisher exact probability test according to data characteristics. Multivariable exact logistic regression analysis was employed to identify the risk factors of POUR. The study size was calculated based on the 10 events-per-variable requirements for the logistic regression analysis [10]. Considering six potential variables, including age, sex, comorbidities, opioid usage, anticholinergic usage, and pain score, and a POUR prevalence of 41.4%, a total sample of 145 participants was deemed necessary for this study [11]. The threshold for statistical significance was set at *p* < 0.05 and STATA 16 (StataCorp LLC, College Station, Tx, USA) was used for all analyses.

The screening tool was developed according to the transparent reporting of a multivariable prediction model for individual prognosis or diagnosis (TRIPOD) statement [12]. All potential variables were included in multivariable logistic regression modeling under the backward elimination approach. Variables were selected according to statistical significance and clinical relevancy. Subsequently, the scoring system was derived from the final model’s regression coefficient (β). The score performance was assessed using the area under the receiver operating characteristic curve (AuROC). Model calibration was demonstrated with the calibration plot. We performed internal validation using the bootstrap resampling method. Model overfitting was presented with an expected: observed (E: O) ratio, calibration-in-the-large (CITL), and the shrinkage factor.

## Results

A total of 145 patients with a mean age of 80.2 ± 8.1 years were included. Demographic data of all patients are demonstrated in Table 1. Of those, 22 (15.2%) patients had POUR after urinary catheter removal. Patients who had urinary retention after Foley catheter removal had significantly higher IPSS. In addition, current use of drugs with anticholinergic effects and preoperative opioid consumption was considerably higher in the POUR groups. Moreover, the ability to independently get out of bed within 24 h postoperatively was significantly less in the POUR group.

**Table 1:** Patient demographics for fragility hip fracture patients with and without urinary retention after catheter removal

Demographic	Urinary retention	No urinary retention	*p*-value
	(*n* = 22, 15.2%)	(*n* = 123, 84.8%)	
	Mean ± SD	Mean ± SD	
Age (year)	80.9 ± 8.5	80.1 ± 8.1	0.669
Sex (n, %)			0.284
Male	3 (13.6%)	32 (26.0%)	
Female	19 (86.4%)	91 (74.0%)	
BMI^1^ (kg/m^2^)	23.9 ± 3.4	22.3 ± 3.9	0.061
IPSS^2^	11.2 ± 7.1	5.0 ± 2.9	< 0.001
Drug with anticholinergic effect usage (n, %)	5 (22.7%)	4 (3.3%)	0.004
Fracture (n, %)			0.356
Femoral neck fracture	10 (45.5%)	61 (49.6%)	
Intertrochanteric fracture	12 (54.5%)	62 (50.4%)	
Preoperative Hb^3^ (g/dL)	11.6 ± 1.6	11.4 ± 2.0	0.553
Preoperative eGFR^4^	63.5 ± 21.4	70.2 ± 21.2	0.172
Surgery (n, %)			0.816
Hip replacement	9 (40.9%)	47 (38.2%)	
Internal fixation	13 (59.1%)	76 (61.8%)	
Time to surgery (day)	3.9 ± 3.2	3.3 ± 3.4	0.461
Anesthesia (n, %)			1.000
General anesthesia	2 (9.1%)	13 (10.6%)	
Spinal block	20 (90.9%)	110 (89.4%)	
Operative time (minute)	73.1 ± 17.3	67.1 ± 26.8	0.313
Intraoperative blood loss (ml)	185.9 ± 116.1	212.6 ± 143.0	0.409
Time to catheter removal (hr)	41.9 ± 1.8	41.2 ± 3.3	0.367
Residual urine (ml)	467.6 ± 162.5	118.3 ± 72.7	< 0.001
Morphine usage (mg)			
Preoperative	13.0 ± 5.9	9.6 ± 5.4	0.009
Postoperative day 1	4.9 ± 4.4	3.9 ± 2.3	0.135
Postoperative day 2	4.5 ± 3.9	4.1 ± 3.9	0.661
Total	62.6 ± 41.2	47.0 ± 29.2	0.033
Postoperative UTI^5^	3 (23.1%)	5 (7.0%)	0.103
Time to ambulation (day)	4.3 ± 2.2	2.2 ± 1.0	< 0.001
Inability to get out of bed within 24 h after surgery (n, %)	22 (100%)	91 (75.3%)	0.008

^1^BMI, Body Mass Index; ^2^IPSS, International Prostate Symptom Score; ^3^Hb, Hemoglobin; ^4^eGFR, estimated Glomerular Filtration Rate (mL/min/1.73 m^2^); ^5^UTI, Urinary tract infection

**Fig. 1:**
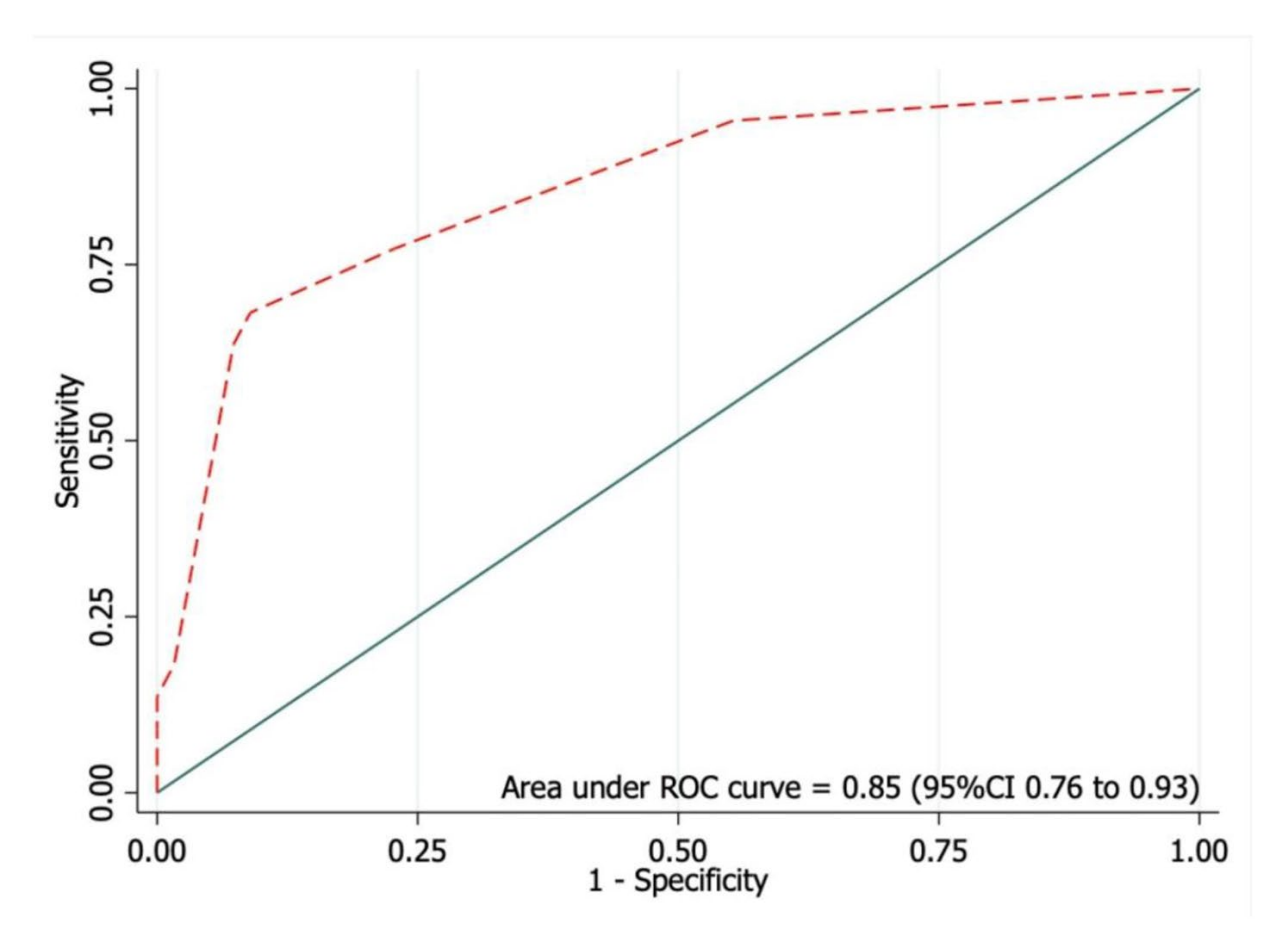
The area under the receiver operating characteristic curve (AuROC) of the predictive

**Fig. 2:**
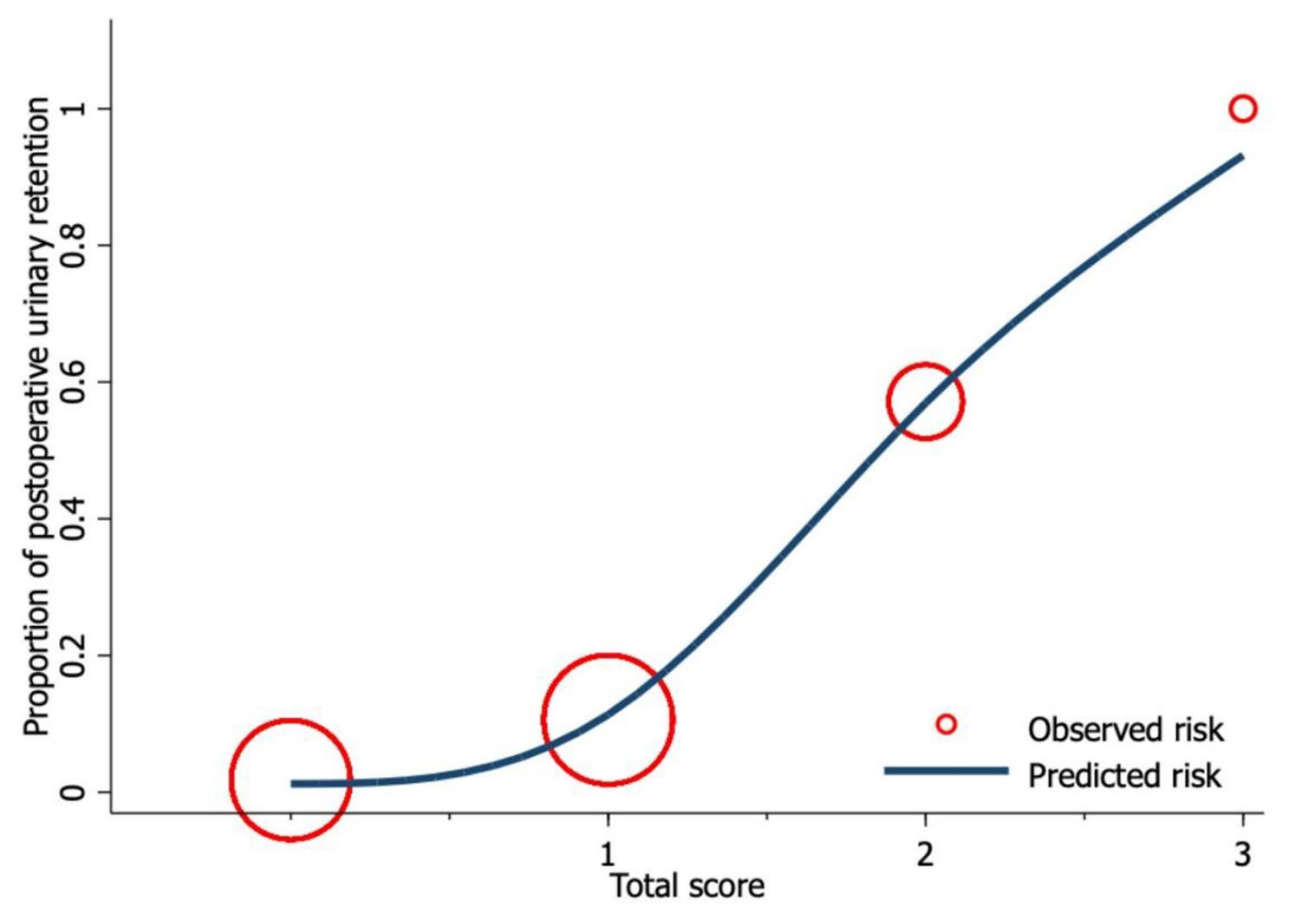
The calibration plot demonstrates an agreement between the predicted risk (navy line) from the predictive score and the observed risk (red circle) for POUR

Univariable exact logistic regression analysis identified current use of drugs with anticholinergic effects (OR = 8.53, *p* = 0.009), IPSS ≥ 8 (OR = 8.67, *p* < 0.001), total morphine consumption (OR = 1.01, *p* = 0.049), and the inability to get out of bed within 24 h after surgery (OR = 9.71, *p* = 0.008) as risk factors for POUR (Table 2). A stepwise backward elimination approach was applied to multivariable logistic regression modeling to create a high-performance screening tool with the smallest number of variables. As a result, the total morphine consumption was eliminated due to the small effect size. The final model included current use of drugs with anticholinergic effects (OR = 11.93, *p* = 0.012), IPSS ≥ 8 (OR = 9.31, *p* < 0.001), and inability to get out of bed within 24 h after surgery (OR = 6.51, *p* = 0.051). The regression coefficient (β) of each variable was then derived into a weighted score (Table 3), which demonstrated good performance with AuROC = 0.83 (95%CI 0.75 to 0.91) (Fig. 1) and good calibration (Fig. 2). In addition, the developed scoring system demonstrates good internal validity with an E: O ratio = 1.03, CITL = 0.02, and the shrinkage factor = 0.95.

**Table 2:** Univariable and multivariable exact logistic regression of fragility hip fracture patients’ postoperative urinary retention variables

Potential variables	uOR	*p*-value	mOR	*p*-value
Age ≥ 80 years	1.46	0.593		
BMI^1^ ≥ 25 kg/m^2^	1.27	0.827		
Medication with anticholinergic effect	8.53	0.009	11.93	0.012
IPSS^2^ ≥ 8	8.67	< 0.001	9.31	< 0.001
Preoperative Hb^3^ < 10 g/dL	1.42	0.708		
Preoperative eGFR^4^ < 60	1.71	0.369		
Intraoperative blood loss (per ml)	0.99	0.420		
Operative time (per minute)	1.01	0.316		
Spinal block	2.79	0.224		
Duration of catheterization (per hours)	1.11	0.361		
Postoperative UTI^5^	3.87	0.206		
Total morphine consumption (per mg)	1.01	0.049		
The ability to independently get out of bed within 24 h	9.71	0.008	6.51	0.051

^1^BMI, Body Mass Index; ^2^IPSS, International prostate symptom score; ^3^Hb, Hemoglobin; ^4^eGFR, estimated Glomerular Filtration Rate (mL/min/1.73 m^2^); ^5^UTI, Urinary tract infection

**Table 3:** Multivariable exact logistic regression after backward elimination with transformed coefficients and assigned score

Variables	multivariable analysis		Score	
	β	p-value	Transformed β	Assigned score
Medication with anticholinergic effect	2.48	0.012	1.37	1
IPSS^1^ ≥ 8	2.23	< 0.001	1.23	1
Inability to get out of bed within 24 h after surgery	1.87	0.051	1.00	1

^1^IPSS, International prostate symptom score

For practicality, we categorized the weighted score into high- and low-risk groups regarding the diagnostic performance of the cut-off level. Accordingly, a cut-off level of 2 demonstrated high specificity (81.7%) and negative predictive value (95.1%) to identify patients who are at risk for POUR and might benefit from delayed urinary catheter removal (Table 4).

**Table 4:** The developed prediction risk categories with sensitivity, specificity, PPV^1^, and NPV^2^

Risk categories	Score	Urinary retention		No urinary retention		Sensitivity	Specificity	PPV^1^	NPV^2^
		n	(%)	N	(%)				
Low risk	< 2	5	22.7	98	81.7	77.3%	81.7%	43.6%	95.1%
High risk	≥ 2	17	77.3	22	18.3				

PPV^1^ positive predictive value, NPV^2^ negative predictive value

## Discussion

The present study demonstrates that the current use of drugs with anticholinergic effects, IPSS ≥ 8, and the inability to get out of bed within 24 h after surgery are significant variables for POUR after urinary catheter removal in fragility hip fracture patients. In addition, the screening tool developed from these variables demonstrates good performance and minimal overfitting.

Previous studies found that POUR was common among geriatric hip fracture patients. Although the incidence ranged from 11.1 to 41.4%, and particularly 88% of POUR was asymptomatic [11, 13, 14]. Failure to determine patients’ risk of POUR leaded to improper urinary catheter removal and might require a catheter re-insertion [11]. Furthermore, POUR significantly increased the UTI-induced sepsis rate in older patients [15]. Accordingly, identifying risk factors of POUR in geriatric hip fracture patients is, therefore, essential in preventing POUR and its related complications [14].

Our discovered risk factors of POUR are consistent with previous findings [16]. Current use of anticholinergic drugs and opioid consumption are associated with urinary retention. Medications with anticholinergic effects, such as antipsychotics, tricyclic antidepressants, and antihistamine medications are recognized for their association with acute urinary retention by inhibiting detrusor muscle contraction via the parasympathetic pathway [16, 17]. Opioids and their derivatives partially inhibit parasympathetic activity while increasing sphincter tone via the sympathetic pathway [18]. Hence, anticholinergic medication avoidance, as well as an appropriate perioperative morphine prescription, is recommended to reduce the risk of perioperative urinary retention in older patients with hip fractures.

IPSS was initially developed to determine the severity of LUTS in males [7]. Nevertheless, two studies demonstrated that IPSS could accurately determine the LUTS severity in females [19, 20]. IPSS comprises seven questions related to voiding symptoms. A score of 0 to 7 indicates mild, 8 to 19 indicates moderate, and 20 to 35 indicates severe symptoms [7]. Our study demonstrated that patients with moderate and severe LUTS determined by IPSS were at risk for POUR after hip surgery. Therefore, these particular patients may benefit from further investigation of their urological symptoms.

Patients with fragility hip fractures usually are unable to ambulate as a result of pain from the time of injury through perioperative periods. Consequences of immobilization including pressure injury, delirium, and POUR have been reported in the literature [21]. Several measures have been proposed in order to reduce immobilization and enhance recovery, including adequate perioperative pain control, expedited hip fracture surgery, prevention of delirium and early mobilization. Similar to our finding, the ability to ambulate out of bed within 24 h after the operation is associated with a lower rate of urinary retention after urinary catheter removal. Therefore, postoperative early ambulation on the next day is strongly recommended in order to prevent the complication of immobilization and lower the risk of perioperative urinary retention. However, it is reasonable to delay catheter removal in patients who are unable to do early ambulation within the first 24 h after surgery in order to prevent POUR [22]. Furthermore, urinary retention after catheter removal should be detected earlier by using a bladder scan, especially in high-risk patients [23].

The developed screening tool could identify patients at risk for POUR. Patients with scores of 2 and above are categorized as high risk and need close monitoring after Foley catheter removal to prevent asymptomatic POUR. This particular group of patients might benefit from delayed urinary catheter removal. In addition, patients identified as high risk based on our screening tool should be provided with preventive measures, including medication reconciliation, perioperative pain management, implementation of an enhanced recovery after surgery (ERAS) protocol, and adherence to a multidisciplinary rehabilitation protocol by the care team.

There are several strengths in this study. the diagnosis of POUR in this study is reliable since all patients underwent an ultrasonography bladder scan from an experienced urologist. There were no missing data and patient dropouts in this study. Moreover, this study successfully developed a high-performance screening tool based on a prospective study. However, there are some limitations in this study. First, although the quality of the data collection in this study is high due to the prospective data collection, the result of this study might not be applicable to certain patients who were not eligible for inclusion, such as those with high-energy trauma, individuals requiring postoperative urine monitoring, and patients with a history of pelvic radiation. Second, some variables in the screening tool were unmodifiable (e.g., IPSS and previous medication), which might limit the utility of the screening tool. Third, some potential preoperative predictors, such as preoperative post-void urinary volume, were not feasible to collect. Fourth, we did not use a clinical outcome such as ‘painful retention’ as the specific outcome of the present study. Although the residual urine is generally accepted as a surrogate measurement for urinary retention, its clinical correlation is limited. Finally, although the screening tool demonstrated good performance. The screening tool elucidated from this cohort has not been validated or tested outside the developmental cohort. Therefore, external validity testing of the score is mandatory before clinical use.

## Conclusions

We identified medications with anticholinergic effects, opioid consumption, IPSS equal to or greater than 8, and the inability to get out of bed within 24 h after surgery as significant risk factors for POUR in fragility hip fracture patients. The risk of POUR could be minimized by avoiding medicine associated with POUR while promoting early ambulation in surgically treated fragility hip fracture patients. A scoring system for POUR screening was developed and proposed for further external validation.

## Data Availability

The datasets generated and/or analyzed during the current study are not publicly available due to ethical reasons but are available from the corresponding author on reasonable request.

## Abbreviations

POUR: Postoperative urinary retention
ERAS: Enhanced recovery after surgery
LUTS: Lower urinary tract symptoms
IPSS: International prostate symptom scores
UTI: Urinary tract infection
TRIPOD: Transparent reporting of a multivariable screening model for individual prognosis or diagnosis
VAS: Visual Analog Scale
CITLI: calibration-in-the-large
AuROC: Area under the receiver operating characteristic curve
